# Oxidative stress and growth performance are modulated by polyunsaturated fatty acids in Arctic charr, brook charr and their reciprocal hybrids

**DOI:** 10.1093/conphys/coaf032

**Published:** 2025-05-28

**Authors:** Pierre U Blier, Grant W Vandenberg, Nathalie R Le François, Emilie Proulx, Francis Dupuis, Moïse Cantin, Véronique Desrosiers, France Dufresne, Felix Christen

**Affiliations:** Département de Biologie, Université du Québec à Rimouski, 300 Allée des Ursulines, Rimouski, QC, G5L3A1, Canada; Département des Sciences Animales, Pavillon Paul-Comtois, 2425 rue de l'Agriculture, Université Laval, Québec, QC G1V 0A6, Canada; Division des Collections Vivantes, Conservation et Recherche, Biodôme de Montréal/Espace pour la vie, Laboratoire de Physiologie, Aquaculture et Conservation, 4777 Av. Pierre-De Coubertin, Montréal, QC H1V 1B3 Montréal, QC H1V 1B3, Canada; Département des Sciences Animales, Pavillon Paul-Comtois, 2425 rue de l'Agriculture, Université Laval, Québec, QC G1V 0A6, Canada; Aquaculture Gaspésie Inc, 1811 Mnt de Pointe Navarre, Gaspé, QC G4X 1A8, Canada; Pisciculture des Monts-de-Bellechasse, 251 Rue Commerciale, Saint Damien-de-Buckland, QC G0R 2Y0, Canada; Département de Biologie, Université du Québec à Rimouski, 300 Allée des Ursulines, Rimouski, QC, G5L3A1, Canada; Département de Biologie, Université du Québec à Rimouski, 300 Allée des Ursulines, Rimouski, QC, G5L3A1, Canada; Département de Biologie, Université du Québec à Rimouski, 300 Allée des Ursulines, Rimouski, QC, G5L3A1, Canada

**Keywords:** Fatty acids, growths, oxidative, performance, pisciculture, salmonids, stresses

## Abstract

In fish, polyunsaturated fatty acids (PUFAs) are essential structural elements in cellular membranes, participate in pathway regulation and act as important energy storage sources for optimum growth performance. However, they are also highly susceptible to peroxidation and thus potential oxidative damage. Omega-3 fatty acid content can vary among individuals and populations of fish and can therefore modulate their health status or resistance to oxidative stress. Our objective was to modulate $ \Sigma $ omega-3 content in fish through different diets and estimate its impact on growth performance, overall fatty acid composition, oxidative stress parameters and antioxidant activity. We conducted experiments on juveniles (1^+^) of four salmonid groups: Arctic charr (*Salvelinus alpinus),* brook charr (*Salvelinus fontinalis*) and their reciprocal hybrids. We found that growth performance in the four groups was negatively affected by high dietary fatty acid content. The content of thiobarbituric acid reactive substances (TBARS, a marker of lipids peroxidation) significantly rose in Arctic charr when fed the omega-3-rich diet. It was also observed that individuals with high docosahexaenoic acid and low $ \Sigma $ omega-6 content had lower TBARS content. Consequently, high omega-3/omega-6 ratios were accompanied by lower oxidative stress levels. This supports the utilization of omega-3/omega-6 ratios as a marker of the ability of fish to modulate oxidative stress both in the wild and in an aquaculture context. This will further help to predict responses to environmental or nutritional modifications.

## Introduction

In fish, polyunsaturated fatty acids (PUFAs) are an essential structural element for cellular membranes and are involved in different cellular pathways and chromatin regulation (Papsdorf and Brunet, 2019). They are needed for optimum growth performance (Tocher, 2010), and the requirements of essential fatty acids may differ between different life stages of fish, such as larval or juvenile stages (Eryalçın *et al.,* 2013, 2015; Tseng *et al.,* 2023) or broodstock (Pickova *et al.*, 2007). Deficiencies in PUFAs through inadequate feeding regimes can therefore affect different processes throughout these life stages (restricted growth performance, perturbations of reproductive processes). These restrictions have also been associated with numerous pathologies that may ultimately cause the death of an individual (Das, 2006)). Fish PUFAs, and especially omega-3 (ω-3) fatty acids, provide an essential nutritional requirement for humans and are beneficial to their health (Calder, 2014). Humans rely on fish and other marine products as their principal source of long chain ω-3 fatty acids (Calder and Yaqoob, 2009). The first accounts of the beneficial effects of ω-3 on human health came from observations in indigenous people from Greenland where, despite a high-fat diet, incidence of cardiovascular diseases was extremely low likely due to increased dietary ω-3 intake (Bang *et al.,* 1971). Since then, ω-3 fatty acids have demonstrated benefits in attenuating Alzheimer’s disease and depression (Hallahan *et al.,* 2016; Song *et al.,* 2016), as well as in cardiovascular health (Kris-Etherton *et al.,* 2002) and chronic disease management (Lordan *et al.,* 2011), and they also exhibit anti-inflammatory activities via their metabolites (Wall *et al.,* 2010). Therefore, there is a growing interest in the aquaculture industry to produce fish species or strains with high levels of these fatty acids. Dietary fatty acid composition is readily reflected in fish muscle, and diets can be altered to enhance ω-3 deposition in fish (Bell *et al.,* 2001; Jobling, 2004). However, there are limits to increasing ω-3 content in the flesh of aquaculture fish. Due to their chemical structure, PUFAs (as ω-3) are extremely sensitive to oxidative processes and therefore oxidative stress. The consequence can be accumulation of damage to DNA, proteins and membrane lipids (Halliwell and Gutteridge, 2015). Increased dietary ω-3 content has been shown to increase susceptibility to peroxidation and oxidative damage of tissue lipids in *Scophthalmus maximus* (Stéphan *et al.,* 1995)*.* Despite their essential role, excess ω-3 could be detrimental to fish health due to an increased risk of peroxidation (Hsieh and Kinsella, 1989). For instance, in Arctic charr (*Salvelinus alpinus)* and the gibel carp (*Carassius auratus gibelio*), an ω-3-rich diet induced significant decreases in growth performance of the two species (Olsen and Henderson, 1997; Chen *et al.,* 2011). In a previous study (Christen *et al.,* 2020) we also observed that fatty acid profiles in heart tissues of Arctic charr, brook charr and their hybrids are associated with fish tolerance to temperature increase (CTmax), suggesting a functional link between this profile and fish robustness. This supports the assumption that fatty acid profiles in key tissues could be a powerful marker of health status (Oliva-Teles, 2012) and tolerance to habitat changes. In a conservation context, oxidative stress markers have emerged as valuable tools for evaluating health status, individual survival and reproductive potential, and ultimately, the impact of human activities on populations (Beaulieu and Costantini, 2014; Blier, 2014). Recognizing that factors such as membrane composition, particularly fatty acid composition, can impact the oxidative stress output and the significance of these markers (Christen *et al.,* 2020); incorporating this trait could refine the precision of these tools for diagnosing individual health and predicting future outcomes of individuals or populations.

This study aimed to investigate how varying dietary ω-3 fatty acid levels affect growth performance, fatty acid profile, oxidative stress parameters and antioxidant capacity in four groups of salmonids. As fish species vary greatly in their fatty acid content and utilization capacities (Glenncross *et al.,* 2014; Murray *et al.,* 2014), we chose two species of salmonids that are ecologically and economically important in many northern countries: Arctic charr (AC, *S. alpinus*) and brook charr (BC, *Salvelinus fontinalis*). Additionally, we used their respective hybrids AC ♀ × BC ♂ (HA) and AC ♂ × BC ♀ (HB). Hybrids in general have a great potential to positively influence productivity of fish farms by reducing inbreeding and improving growth performance (Bartley *et al.,* 2000; Blackie *et al.,* 2011). Furthermore, hybridization of these species has been observed in the wild (Hammar *et al.,* 1991; Bernatchez *et al.,* 1995), and the hybrids AC × BC and AC (Fraser) × AC (Nauyuk) are both currently reared at one of our industrial partner’s facilities (Pisciculture des Monts-de-Bellechasse Inc. and Aquaculture Gaspésie Inc., respectively). Hybridization may however induce the disruption of mitonuclear coadaptation and exacerbate oxidative stress by loss of mitochondrial functional integrity (Pichaud *et al.,* 2019). This might amplify a potential negative impact of an ω-3-rich diet on the physiological integrity of individuals. The four groups were fed an ω-3-rich (WW) and a control diet (W) for a 140-day growth period. Following the growth trial, fatty acid profiles of muscle tissue, oxidative stress levels (thiobarbituric acid reactive substances [TBARS], protein carbonyls) and antioxidant activity (catalase [CAT] and superoxide dismutase [SOD]) were determined. We hypothesized that growth performance would be negatively affected in all four groups fed the WW diet. Furthermore, we predicted that this would result in an increase in oxidative stress markers due to a higher peroxidation index (PI) associated with the increased ω-3 PUFA content. Endogenous antioxidant activity is known to be affected by dietary lipid content in rainbow trout (*Oncorhynchus mykiss*; Fontagné-Dicharry *et al.,* 2014). Therefore, we wanted to test if groups or individuals with higher ω-3 content could prevent adverse impact due to susceptibility to oxidative stress, by compensation of antioxidant activity. Subsequently, a breeding programme selecting fish with high levels of antioxidant capacity, or able to respond to the high level of ω − 3 by mobilizing antioxidant mechanisms, could be initiated. Moreover, we hypothesized that hybrids could be more impacted by high ω-3 content than parental species, since hybridization of two species can lead to loss of co-adaptation between nuclear and mitochondrial genomes (Blier *et al.,* 2001), therefore amplifying oxidative stress and revealing its association with fatty acid profiles and growth performance.

## Materials and Methods

### Fish

Arctic charr (*S. alpinus,* AC, Fraser strain), brook charr (*S. fontinalis,* BC, Baldwin strain) and two hybrids, AC ♀ × BC ♂ (HA) and AC ♂ × BC ♀ (HB), were provided by Pisciculture des Monts de Bellechasse Inc. (St-Damien-de-Buckland, QC, Canada) and Aquaculture Gaspésie Inc. (Gaspé, QC, Canada). Mean fish mass was 422 ± 55 g (AC), 489 ± 39 g (BC), 303 ± 51 g (HA) and 272 ± 40 g (HB). Growth experiments were conducted at the Laboratoire Régional des Sciences Aquatiques (LARSA; Université Laval, Québec, QC, Canada). Fish were randomly distributed in 300-l tanks supplied with 99% recirculating water at 10.5°C under natural photoperiod. Fish were distributed in three tanks per species or line (20 fish per tank). After a 4-week acclimation period, all individuals were weighed and identified subcutaneously using passive integrated transponders (PIT-Tag, Biomark, ID, USA). All experiments were authorized by the local animal ethics committee (CPA Université Laval).

### Feeding and diets

Weekly feeding schedule for a 140-day growth period was as follows: fish were hand-fed to apparent satiety twice a day for 2 days, fed to 80% of satiety twice daily for the following 4 days and starved for 24 h. Fish were fed two different diets, one with a fatty acid composition close to commercial feeds (W) and one with increased $ \Sigma $ omega-3 fatty acid content (WW). For each dietary treatment, 12 tanks with fish were used (3 per species or line). Diets were mixed, steam pelleted using a California Pellet Mill (Model CPM CL-5, Crawfordsville, IN, USA), dried overnight in a forced-air oven set at 22°C and stored at 4°C. Pellet size was adjusted throughout the experiment to fit fish size. Diet formulation is presented in Table 1. Fatty acid profiles and proximate composition of both diets are presented in Table 2.

**Table 1:** Diet formulation

Ingredients	g/kg
Herring meal^a^	114
Sardine meal^a^	86
Fish oil^a^^,^^b^	170
HP300 soybean protein^c^	110
Pea protein^d^	130
Corn meal^e^	180
Whole wheat meal^f^	80
Blood meal^g^	100
CaHPO4^c^	20
Vitamins and minerals^h^	9.8
Astaxanthin^i^	0.05

a Swimco Canada, Toronto, ON, Canada.

b Oil type changed for the two treatments, commercial oil (W) and $ \Sigma $ omega-3 enriched oil (WW).

c Jefo, St-Hyacinthe, QC, Canada.

d Parrheim Foods, Saskatoon, SK, Canada.

e Meunerie de St-Frédéric Inc. Saint-Frédéric, QC, Canada.

f La Seigneurie des Aulnaies Inc., Saint-Roch-des-Aulnaies, QC, Canada.

g AP301 spray dried, APC nutrition Inc., Calgary, AB, Canada.

h Corey Nutrition Company, Fredericton, NB, Canada.

i Carophyll pink, DSM nutritional products AG, Kaiseraugst, Switzerland.

**Table 2:** Lipid and proximate composition of the two experimental diets (relative percentages)

	W (low ω-3 content)			WW (high ω-3 content)		
C16:0	14.80	±	0.05	16.76	±	0.04
C18:0	2.02	±	0.02^*^	4.48	±	0.02
SFA	24.58	±	0.12	26.12	±	0.09
C16:1	6.91	±	0.07	7.20	±	0.07
C18:1	13.42	±	0.06	15.98	±	0.08
MUFA	33.91	±	0.19^*^	29.33	±	0.19
C18:2n6	8.77	±	0.20	8.75	±	0.20
C20:4n6 (ARA)	0.40	±	0.00^*^	0.87	±	0.01
n-6	9.63	±	0.21	10.61	±	0.22
C18:3n3	1.76	±	0.04	1.80	±	0.02
C20:5n3 (EPA)	5.86	±	0.04^*^	11.11	±	0.09
C22:5n3 (DPA)	0.77	±	0.01^*^	1.50	±	0.01
C22:6n3 (DHA)	5.94	±	0.08^*^	9.92	±	0.08
n-3	14.83	±	0.10^*^	25.40	±	0.15
n-3/n-6	1.55	±	0.03^*^	2.41	±	0.05
PI	117.18	±	0.88^*^	185.20	±	1.11
UI	147.56	±	0.74^*^	186.12	±	0.80
Moisture (%)	5.54	±	0.58	6.01	±	0.89
Lipid (%)	19.28	±	0.99	20.56	±	0.85
Protein (%)	44.52	±	0.19	46.12	±	0.25
Ash (%)	9.80	±	0.56	10.50	±	0.74

Values are means ± SEM. SFA, saturated fatty acids; n-3N6, $ \Sigma $ omega-3/$ \Sigma $ omega-6 ratio.

^*^Significant differences (*P* < 0.05). Fatty acyl chains with values lower than 1% g are not shown except for ARA.

### Fish sampling

At the end of the growth trial, fish were starved for 3 days, euthanised with a blow to the head, weighed and dissected. Individual organ mass (heart, intestine, liver, pyloric caeca and gonads) and carcass mass were determined. One fish per species or line and tank was kept for proximate analysis. A second fish was filleted and one fillet was kept for proximate analysis, while the red muscle of the other fillet was extracted. The red muscle was homogenized in ice cold phosphate buffer (100 mM, 20 mM EDTA, pH = 8.0), nitrogen flushed, flash frozen in liquid nitrogen and stored at −80°C for subsequent analysis.

### Proximate analysis

Whole fish, fillets and diet were homogenized at 4°C, aliquoted and stored at −80°C for proximate analysis. Proximate analysis was done using standard methods of AOAC (2012). Crude ash content was determined by incinerating samples in a furnace at 550°C overnight. Nitrogen was determined on lyophilized samples using an elemental analyser (Nx6.25). Moisture content was determined by drying the samples at 105°C for 24 h. Crude lipid content was determined by Soxhlet extraction (Soxhlet, 1879).

### Fatty acid profiles of fillets and experimental diets

A protocol adapted from Lepage and Roy (1984) and applied in Ekström *et al.* (2017) was used in the current experiment. At least 100 mg of tissue (*N* = 15) was homogenized (1:5 dilution) in 100 mM potassium phosphate buffer (1 mM EDTA, pH 7.5) spiked with 0.1 mg of internally added tridecanoic and tricosanoic acid (Nu-Check Prep, Elysian MN, USA). Direct acid-catalysed trans-methylation was performed by adding 3 ml of 3% sulphuric acid methanol solution and heating at 90°C for 1 h. To prepare fatty acid methyl esters (FAME), samples were cooled to 4°C, 5 ml of H_2_O and 1 ml of hexane were added and the sample was vortexed and centrifuged at 3000*g* for 10 min at room temperature. Hexane was evaporated, and the sample was suspended in 100-μl toluene prior to injection. FAME were separated and quantified by gas chromatography (Trace Ultra 100, Thermo Fisher Scientific, Waltham, MA, USA) equipped with a 60-m × 0.32-mm i.d. capillary column (DB-23, Agilent Technologies Canada, Mississauga, ON, Canada). Helium was used as a carrier gas (230 kPa constant pressure), and vaporization temperature was set at 230°C with split injection of 100 ml min^−1^. Temperature programming was from 50 to 140°C (25°C min^−1^), 140 to 195°C (3°C min^−1^) and a final increase of 4°C min^−1^ up until 225°C maintained for 5 min. Individual methyl esters were identified by comparison with known standards. All chemicals were purchased from Sigma-Aldrich unless otherwise mentioned.

### Oxidative stress parameters and antioxidant activity

Red muscle was homogenized in 100 mM phosphate buffer at pH 7.0 (*N* = 120, 60 for each diet and 15 per group). Subsequently, one aliquot of the homogenate was centrifuged (4°C) at 1600*g* for 15 min, and the supernatant was used for TBARS analysis. Another one was centrifuged 1500*g* for 10 min (4°C), and the supernatant was used for protein carbonyls determination. Finally, another aliquot was centrifuged 1300*g* for 1 min (4°C), and the supernatant was used for SOD and CAT, respectively.

### Thiobarbituric acid reactive substances

TBARS content was measured by following the controlled reaction of malondialdehyde (MDA) with thiobarbituric acid using an adapted protocol (Ohkawa *et al.,* 1979; Said *et al.,* 2014). Briefly, 50 µl of supernatants were incubated with 375 µl of thiobarbituric acid (0.8%); 50 µl of sodium dodecyl sulphate (8.1%); 375 µl of 20 % acetic acid in aqueous solution (v:v); 150 µl of distilled water at 100°C for 60 min. Adducts formed during the reaction were then quantified using the EnVision multilabel platereader (Perkin Elmer Envision, Foster City, CA, USA) at an excitation wavelength of 540 nm and an emission wavelength of 550 nm. Protein concentration was determined using the Bicinchoninic Acid (BCA) assay (Smith *et al.,* 1985). Results were expressed as nanomoles of MDA per milligram of protein.

### Protein carbonyls

Levels of carbonylated proteins were quantified using the protein carbonyl colorimetric assay kit from Cayman Chemicals (Ann Arbor, MI, USA). Briefly, this assay is based on the reaction between 2,4-dinitrophenylhydrazine and protein carbonyls producing protein hydrozone. The latter is then quantified spectrophotometrically at 370 nm using the EnVision multilabel plate reader (Perkin Elmer Envision).

### Catalase activity

Catalase (CAT) activity was estimated using an adapted protocol from Orr and Sohal (1992). In short, the supernatant was added to a reaction medium containing 60 mM of hydrogen peroxide (H_2_O_2_). The decomposition rate of H_2_O_2_ was then followed by measuring the decrease in absorbance at 240 nm for 90 s using a spectrophotometer Lambda 11 (Perkin Elmer, Foster City, CA, USA). One unit of CAT is defined as the amount of enzyme needed to reduce 1 μmol of H_2_O_2_ min^−1^.

### Superoxide dismutase activity

SOD activity was determined using the superoxide dismutase assay kit from Cayman Chemicals. According to the manual, the assay follows superoxide radicals generated by xanthine oxidase and hypoxanthine using tetrazolium salt for spectrophotometric detection at 450 nm in the supernatant. Total SOD is expressed as means of U mg^−1^ proteins ± SEM, where one unit of SOD is defined as the amount of enzyme needed to exhibit 50% dismutation of the superoxide radical.

### Calculations and statistical analysis

#### Growth performance

Specific growth rate (SGR) was calculated as follows:


$$ SGR=\left[\frac{\left( lnW2- lnW1\right)\ }{t}\right]\ast 100 $$

where *W1* and *W*2 are the body mass at the beginning and the end of the experiment and *t* is the number of days the growth trial lasted. PI and unsaturation index (UI) were calculated according to Hulbert *et al.* (2007):

PI = (0.025 × % monoenoics) + (1 × % dienoics) + (2 × % trienoics) + (4 × % tetraenoics) + (6 × % pentaenoics) + (8 × hexaenoics).

UI = (1 × % monoenoics) + (2 × % dienoics) + (3 × % trienoics) + (4 × % tetraenoics) + (5 × % pentaenoics) + (6 × hexaenoics).

### Statistical analysis

All statistical analyses were performed using R (R Core Team, 2013), a language and environment for statistical computing (R Foundation for Statistical Computing, Vienna, Austria; http://www.R-project.org/). Data were tested for normality (Shapiro–Wilk) and homogeneity of variance (Bartlett) and transformed when needed. Fatty acid profiles, oxidative stress indicators, antioxidant activities, proximate analysis and growth rates were analysed with a linear mixed effects model with the ‘nlme’ package (Pinheiro *et al.*, 2014), treating diet and group as fixed effects with tank as a random effect. Multiple comparisons were then tested by pairwise comparison of Tukey adjusted least square means. Statistical significance was set at *P* < 0.05, and all values are expressed as means ± SEM. Relationships between TBARS content and the different parameters were established using linear regression analysis. Dietary fatty acid profiles were compared using *t*-tests and adjusted *P* values (0.05).

## Results

### Fatty acid composition and proximate analysis

The experimental diets exhibited significant differences in total omega-3 content (∑ omega-3), with the WW diet showing nearly twice the relative content of eicosapentaenoic acid (EPA), docosahexaenoic acid (DHA) and docosapentaenoic acid (DPA) compared to the control. Most other parameters remained unchanged (Table 2). As expected, the fatty acid composition of the W diet was close to commercial fish feed.

It has to be noted that the two diets also varied slightly in monounsaturated fatty acid (MUFA) and octadecanoic acid (C18:0) content. Arachidonic acid (ARA, C20:4n6) was slightly higher in the WW diet, but both values were under 1% of total fatty acid content. There was no significant difference in proximate composition of whole fish or fillet (Table 3). Fatty acid profiles of the dietary treatments were reflected in the lipid composition of muscle tissues (Table 4). The WW diet resulted in higher levels of EPA, DHA, DPA and total $ \Sigma $ omega-3 relative content in all groups. In the WW treatment the EPA ratio in BC was lower than in AC group while in the W treatment it was lower in BC than all other groups. Also, AC had a lower DHA relative content than BC when fed the W diet. The differences in muscle DHA, DPA, EPA and total $ \Sigma $ omega-3 relative content among groups were not as important as between the experimental diets. All groups fed the WW diet had slightly higher total MUFA and C18:0 relative content than those fed the W diet. Total $ \Sigma $ omega-6 relative content was significantly lower in BC than HA in the W treatment. Furthermore, the W diet resulted in BC having a higher ω-3/ω-6 ratio than AC and HA. ω-3/ω-6 ratios did not differ significantly between treatments. Finally, PI and UI were significantly increased in all four groups fed the WW diet.

**Table 3:** Proximate composition (% on wet weight basis) of muscle and whole body of experimental fish

	W				WW			
	AC	BC	HA	HB	AC	BC	HA	HB
Muscle								
Moisture	65.9 ± 0.7	68.4 ± 0.9	67.4 ± 0.8	67.0 ± 1.2	65.1 ± 0.6	66.1 ± 0.6	67.7 ± 0.7	66.6 ± 0.9
Lipid	7.2 ± 0.4	5.9 ± 0.2	5.6 ± 0.4	8.0 ± 0.7	6.9 ± 0.5	5.5 ± 0.4	5.5 ± 0.2	5.6 ± 0.4
Protein	19.0 ± 0.9	19.5 ± 0.9	20.2 ± 0.9	19.7 ± 1.2	18.0 ± 0.5	20.2 ± 0.7	21.0 ± 0.5	19.2 ± 0.9
Ash	1.6 ± 0.1	1.5 ± 0.1	1.6 ± 0.1	1.5 ± 0.1	1.6 ± 0.1	1.4 ± 0.1	1.5 ± 0.1	1.5 ± 0.1
Whole body								
Moisture	64.5 ± 1.5	62.0 ± 0.9	64.5 ± 1.0	64.0 ± 1.1	61.3 ± 1.3	60.3 ± 1.5	65.7 ± 1.0	63.9 ± 1.2
Lipid	10.1 ± 0.7	10.2 ± 0.5	9.8 ± 0.6	10.5 ± 0.7	10.4 ± 0.4	10.6 ± 0.6	10.8 ± 0.7	10.1 ± 0.2
Protein	16.6 ± 1.0	17.7 ± 0.6	16.6 ± 0.8	17.0 ± 0.7	18.6 ± 1.0	20.2 ± 1.0	18.1 ± 0.6	17.1 ± 0.7
Ash	1.9 ± 0.1	1.8 ± 0.2	1.8 ± 0.1	1.7 ± 0.1	1.9 ± 0.1	1.9 ± 0.1	1.8 ± 0.1	1.9 ± 0.1

W is the control group (low ω-3 content diet) and WW is the high ω-3 content diet fed group, with Arctic charr (AC), brook charr (BC), hybrid arctic (HA) and hybrid brook (HB).

**Table 4:** Lipid composition (% of total fatty acids) of fillets of the four experimental groups and two dietary treatments (fatty acyl chains with values lower than 1% are not shown except for ARA and C18:3n3)

	W				WW			
	AC	BC	HA	HB	AC	BC	HA	HB
C16_0	14.11 ± 0.29	13.92 ± 0.26	14.00 ± 0.23	14.26 ± 0.24	14.61 ± 0.22	14.53 ± 0.18	14.21 ± 0.25	14.38 ± 0.21
C18_0	1.8 ± 30.06^a^	1.96 ± 0.05^a^	1.95 ± 0.07^a^	1.91 ± 0.04^a^	2.54 ± 0.07^A^	2.66 ± 0.06^A^	2.55 ± 0.06^A^	2.59 ± 0.08^A^
SFA	26.25 ± 0.85	25.32 ± 0.87	25.23 ± 0.57	28.23 ± 0.40	27.89 ± 0.66	28.95 ± 0.71	28.10 ± 1.04	27.85 ± 0.91
C16_1	3.91 ± 0.32	4.24 ± 0.27	4.26 ± 0.36	4.14 ± 0.32	3.65 ± 0.32	4.41 ± 0.30	4.33 ± 0.28	4.21 ± 0.21
C18_1	9.87 ± 0.94	10.14 ± 0.84	10.20 ± 1.19	9.94 ± 0.92	10.50 ± 0.80	10.42 ± 0.82	10.75 ± 0.49	10.48 ± 0.72
MUFA	22.91 ± 0.94^a^	22.47 ± 1.20^a^	22.23 ± 1.00^a^	23.63 ± 0.91^a^	17.53 ± 0.92^A^	17.48 ± 0.88^A^	18.90 ± 1.18^A^	17.93 ± 0.95^A^
C18_2n6	3.08 ± 0.19^a,b^	2.75 ± 0.13^a^	3.25 ± 0.24^b^	3.11 ± 0.21^a,b^	3.10 ± 0.16^a,b^	2.98 ± 0.21^a,b^	3.33 ± 0.22^a,b^	3.02 ± 0.16^a,b^
C20_4n6 (ARA)	0.81 ± 0.04^a^	0.74 ± 0.03^a^	0.75 ± 0.02^a^	0.78 ± 0.04^a^	1.12 ± 0.03^A^	1.00 ± 0.02^B^	0.98 ± 0.03^B^	1.03 ± 0.02^A,B^
N6	4.20 ± 0.18^a,b^	3.75 ± 0.15^a^	4.48 ± 0.28^b^	4.18 ± 0.21^a,b^	4.54 ± 0.18^a,b^	4.33 ± 0.24^a,b^	4.58 ± 0.25^a,b^	4.45 ± 0.15^a,b^
C18_3n3	0.73 ± 0.05^a,b^	0.65 ± 0.03^a^	0.79 ± 0.06^a,b^	0.76 ± 0.04^a,b^	0.84 ± 0.04^a,b^	0.91 ± 0.07^B^	0.89 ± 0.06^a,b^	0.84 ± 0.03^a,b^
C20_5n3 (EPA)	7.60 ± 0.17^a^	6.17 ± 0.20^b^	7.15 ± 0.14^a^	7.12 ± 0.19^a^	8.73 ± 0.28^A^	7.79 ± 0.23^B^	8.38 ± 0.22^A,B^	8.14 ± 0.19^A,B^
C22_5n3 (DPA)	1.57 ± 0.05^a^	1.79 ± 0.05^a^	1.69 ± 0.08^a^	1.69 ± 0.06^a^	2.15 ± 0.07^A^	2.41 ± 0.07^A^	2.26 ± 0.09^A^	2.34 ± 0.08^A^
C22_6n3 (DHA)	17.88 ± 0.55^a^	20.40 ± 0.72^b^	18.97 ± 0.61^a,b^	19.44 ± 0.82^a,b^	21.95 ± 0.55^A,B^	23.20 ± 0.73^A^	21.33 ± 0.85^B^	22.03 ± 0.63^A,B^
N3	28.85 ± 0.57^a^	30.12 ± 0.80^a^	29.89 ± 0.53^a^	29.97 ± 0.88^a^	35.18 ± 0.57^A^	35.96 ± 0.62^A^	34.62 ± 0.82^A^	34.95 ± 0.67^A^
N3/N6	7.05 ± 0.34^a^	8.19 ± 0.39^b^	7.04 ± 0.48^a^	7.50 ± 0.48^a,b^	7.93 ± 0.34^a,b^	8.65 ± 0.51^a,b^	7.90 ± 0.48^a,b^	7.98 ± 0.29^a,b^
PI	216.48 ± 4.45^a^	228.57 ± 6.21^a^	224.47 ± 4.23^a^	226.50 ± 6.80^a^	261.13 ± 4.15^A^	267.12 ± 5.00^A^	255.72 ± 6.17^A^	259.67 ± 5.11^A^
UI	192.87 ± 2.94^a^	200.29 ± 4.04^a^	198.89 ± 2.60^a^	200.69 ± 4.15^a^	223.61 ± 2.70^A^	228.06 ± 3.16^A^	221.20 ± 3.90^A^	222.88 ± 3.57^A^
N	15	14	15	15	15	14	15	15

Values are means ± SEM. SFA, saturated fatty acids; N3/N6, omega-3/omega-6 ratio; AC, Arctic charr; BC, brook charr; HA, hybrid arctic; HB, hybrid brook.

Dissimilar letters indicate significant differences between groups; differences in uppercase and lowercase format indicate significant difference between dietary treatments (*P* < 0.05).

### Growth rates and oxidative stress parameters

**Figure 1:**
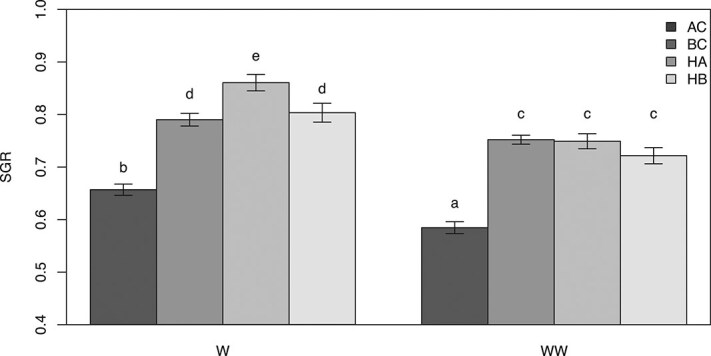
SGRs for all four groups in the two experimental dietary treatments. With Arctic charr (AC), brook charr (BC), hybrid Arctic (HA), and hybrid brook (HB). Different letters indicate significant differences (*P* < 0.05). Values are mean ± SEM.

**Figure 2:**
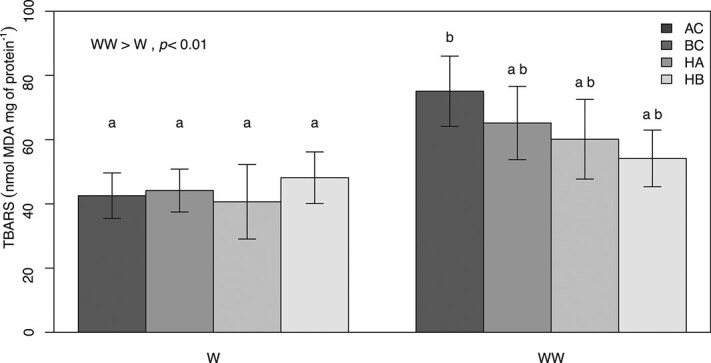
Differences in TBARS content between dietary treatments and groups (W, standard ω-3 content diet, and WW, high ω-3 content diet). With Arctic charr (AC), brook charr (BC), hybrid Arctic (HA), and hybrid brook (HB). Different letters indicate significant differences (*P* < 0.05). Values are mean ± SEM.

**Figure 3:**
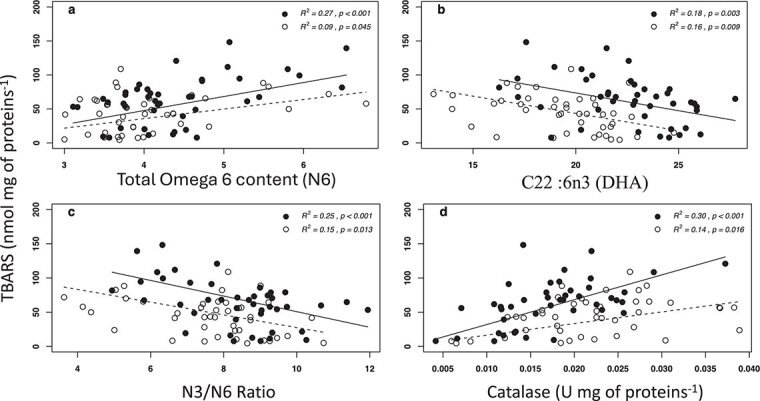
Relationships between TBARS content, total $ \Sigma $ omega-6 content (a), DHA (b), N3/N6 ratio (c) and catalase activity (d) for the two dietary treatments. With W (standard ω-3 content diet) in empty circles and dashed regression lines and WW (high ω-3 content diet) with closed circles and solid regression lines. Equation for regression lines: (**a**) solid (WW): TBARS = 22.43*x* − 35.5; dashed (W): TBARS = 9.88*x* + 4.00; (**b**) solid (WW): TBARS = −5.4*x* + 184.34; dashed (W): TBARS = −3.96*x* + 121.06; (**c**) solid (WW): TBARS = −10.96*x* + 154.42; dashed (W): TBARS = −6.17*x* + 92.36; and (**d**) solid (WW): TBARS = 2983.23*x* + 11.87; dashed (W): TBARS = 1249.14*x* + 16.91.

SGRs decreased significantly for all four groups when fed with the WW diet (Fig. 1). AC had the lowest growth rates in both treatments. HA had the highest mean SGR values when fed the W diet but did not differ in growth performance compared to HB and BC in the WW treatment. TBARS content only increased significantly for AC when fed the WW diet (Fig. 2). There were no differences in protein carbonyl content, superoxide dismutase and catalase activity between groups or treatments (data not shown). TBARS were positively correlated to total $ \Sigma $ omega-6 content and catalase activity (Fig. 3a and d). In both cases, the relationship was stronger for fish fed the WW diet. In contrast, TBARS were negatively correlated to DHA content and the ω-3/ω-6 ratio (Fig. 3b and c).

## Discussion

There has been considerable effort in the aquaculture industry to enhance $ \Sigma $ omega-3 content in fish to increase their value (Trushenski and Bowzer, 2013). This is related to the health benefits of omega-3 fatty acids. For example, DHA and EPA have been shown to protect against oxidative stress by inducing heme-oxygenase 1 (HO-1) and catalase through nuclear factor erythroid 2-related factor 2 (Nrf2) activation (Tatsumi *et al.,* 2019). On the other hand, omega-3 PUFAs are highly susceptible to peroxidation, leading to potential oxidative damage, especially at high body temperatures in fish (Madeira *et al.,* 2016). Therefore, the health impacts of PUFAs may depend on their relative content and environmental conditions, particularly in ectotherms. The fatty acid profiles of fish, and particularly the relative content of PUFAs, could serve as a valuable marker for assessing the health status of fish populations and their susceptibility to environmental changes, such as temperature fluctuations. Our study highlights a negative effect of increased dietary omega-3 fatty acid content on growth performance in two salmonid species and their reciprocal hybrids. Increased oxidative stress was observed as one of the negative consequences of diets enriched with high levels of omega-3 fatty acids, particularly in Arctic charr. Our results also demonstrate significant interindividual variability in both omega-3 fatty acid content and the management of oxidative stress. Importantly, increased antioxidant activity was not correlated with increased dietary omega-3 fatty acid content, but a steeper positive correlation between catalase activity and TBARS was observed for fish fed on a high omega-3 diet.

### Fatty acid profiles

The fatty acid composition of fish accurately mirrored the composition of their experimental diets, a well-established phenomenon in fish nutrition (Henderson and Tocher, 1987; Wang *et al.,* 2010). Specifically, fish fed the WW diet had significantly higher levels of omega-3 fatty acids. However, while there was a clear difference in the overall omega-3 content between fish fed the different diets, there was less variation in omega-3 levels among individual fish within each treatment group compared to the variation in the experimental diets themselves. This phenomenon of tightly regulated fatty acid profiles in salmonids, crucial for membrane structure and cellular function, has been well documented (Yang and Dick, 1994; Tocher, 2003). While the WW treatment consistently resulted in higher omega-3 fatty acid levels, this study revealed, for the first time, significant intragroup variation in EPA and DHA content within each dietary treatment across both species and their hybrids. The observed differences in EPA and DHA content between AC and BC within each treatment might reflect dietary and metabolic adaptations to their specific environments (Sargent *et al.,* 1999). Notably, hybrids exhibited higher EPA content than BC when EPA supply was limited, but this difference diminished when dietary EPA levels increased. This suggests that, compared to BC, hybrids may more readily deposit EPA, or else metabolise it differently.

Notably, the hybrid AC♀ × BC♂ (HA) exhibited superior growth performance (SGR) in the W treatment compared to the other hybrid (AC ♂ × BC ♀ [HB]) and both parental species. This finding is of particular interest as hybrids represent a promising avenue for enhancing growth performance and genetic variability in aquaculture broodstocks. Hybrid vigour, where hybrids outperform both parents, is a well-established phenomenon in aquaculture (Bartley *et al.,* 2000). In a previous study, the same hybrid species also demonstrated superior growth performance during the initial phase of a growth trial (Dupont Cyr *et al.,* 2018), although this advantage was not sustained throughout the entire experiment (the first 30 days of a 280-day growth period). However, the superior SGR of HA in the W treatment was not maintained as growth rates for all groups decreased in the WW treatment. Concurrently, TBARS levels significantly increased in the AC group and tended to be higher in the other groups when fed the WW diet. These findings align with previous research demonstrating the negative effects of high dietary PUFA content on growth performance (Olsen and Henderson, 1997; Chen *et al.,* 2011). This association might be attributed to the increased oxidative stress associated with elevated $ \Sigma $ omega-3 levels and lipid peroxidation, as observed in this treatment. High levels of oxidative stress markers have been linked to decreased resistance to pathogens, impaired growth and reduced nutritional quality of fish flesh (Sutton *et al.,* 2006). Other studies have also reported decreased overall health and growth performance in fish (*C. auratus gibelio* and *S. alpinus*) fed high levels of PUFAs (Olsen and Henderson, 1997; Chen *et al.,* 2011). While variations in the ratios of EPA, DHA and ARA are known to influence fitness-related traits in fish, with each species having an optimal ratio (Sargent *et al.,* 1999), the observed differences in these ratios between treatments were not considered a significant factor in the observed growth performance.

We hypothesized that hybrids might exhibit higher TBARS levels due to potential loss of co-adaptation between mitochondrial and nuclear genomes, leading to increased oxidative stress (Barreto and Burton, 2013). However, protein carbonyl levels, another indicator of oxidative stress, did not increase in the WW treatment, suggesting that oxidative damage may be more pronounced in lipids than in proteins. Furthermore, activity of superoxide dismutase, an important antioxidant enzyme, was not significantly influenced by the dietary treatment, nor did it vary between groups. This suggests that this antioxidant defence mechanism may be sufficient or is not required to counteract the oxidative stress induced by the increased dietary omega-3 content.

### Oxidative stress related parameters

Correlation analysis revealed significant relationships between TBARS levels and several factors, including DHA, total omega-6 content, omega-3/omega-6 ratio and catalase activity. In both treatment groups, individuals with a higher relative omega-6 content exhibited greater oxidative damage, as indicated by increased TBARS levels. Conversely, individuals with higher DHA levels appeared to experience less oxidative stress, even in the presence of elevated TBARS levels observed in the WW treatment. This is counterintuitive, as the extreme sensitivity to peroxidation of PUFAs should increase oxidative stress markers levels as mentioned previously and as seen in the relationship of TBARS and ω-6. DHA has been reported to be an essential fatty acid throughout the life cycle of Arctic charr (Murray *et al.,* 2014). A lower oxidative stress level for individuals with high DHA is in accordance with DHA’s essential role. For example, a study reported that increased oxidative stress levels were induced by higher $ \Sigma $ omega-6 content and suppressed by increased $ \Sigma $ omega-3 content in juvenile chinook salmon (Welker and Congleton, 2003). Metabolites of omega-6 fatty acids are primarily associated with pro-inflammatory effects (Ricciotti and FitzGerald, 2012), while the targeted degradation of omega-3 fatty acids primarily yields anti-inflammatory molecules (Tai and Ding, 2010). Increased production of pro-inflammatory components has recently been associated with increased oxidative stress and various human diseases (Das, 2006; Byrne, 2010). It is thus possible that individuals with high $ \Sigma $ omega-6 and low $ \Sigma $ omega-3 content are more prone to oxidative stress and poorer general health. These relationships are reflected in the correlation between TBARS levels and $ \Sigma $ ω-3/$ \Sigma $ ω-6 ratios: the higher the ratio, the lower the oxidative stress incidence. Levels of oxidative stress markers appear to be correlated with animal general health status and have recently been advanced as a major tool to assess fish health with easy to use and standardized procedures (Blier, 2014). For humans, a large body of research is promoting high $ \Sigma $ ω-3/$ \Sigma $ ω-6 ratios to improve general health status (reviewed in Simopoulos, 2002; Simopoulos, 2008). These trends are well reflected in the present correlation between $ \Sigma $ ω-3/$ \Sigma $ ω-6 and TBARS, where individuals with high TBARS levels (likely associated with poorer overall health status) also have lower $ \Sigma $ ω-3/$ \Sigma $ ω-6 ratios. Certainly, future research needs to test whether or not such correlations persist with additional oxidative stress markers.

While we observed a positive correlation between catalase activity and TBARS levels, suggesting a compensatory response to increased oxidative stress, our results also highlight the complex nature of antioxidant defence mechanisms. The upregulation of catalase, although beneficial, was not sufficient to fully protect against the oxidative damage induced by the pro-oxidative diet, indicating that additional factors may be involved in determining the overall oxidative stress status of the fish. The relationship between increased antioxidant enzyme activity and higher oxidative stress values is largely documented in fish (Tocher *et al.,* 2003; Lesser, 2006; Fontagné *et al.,* 2008). Most studies in this field report results as mean values for different groups, species, or dietary treatments. However, studies that leverage interindividual variability to identify correlations, as in the present study, are rare. Recent research emphasizes the importance of investigating the mechanisms underlying interindividual differences. In a related study, we established a link between ARA and tolerance to high temperature by examining interindividual variation (Christen *et al.,* 2020). We also demonstrated that at a given temperature, individuals with less efficient mitochondrial function produce more reactive oxygen species, increasing their susceptibility to oxidative stress (Christen *et al.,* 2018).

## Conclusion

In summary, this study has shown that growth rates of Arctic charr, brook charr and their respective hybrids are all negatively affected by high dietary content of $ \Sigma $ omega-3. Concomitantly, at least for one group, oxidative stress indices expressed a higher amount of TBARS associated with high $ \Sigma $ omega-3 diet. Additionally, extensive interindividual variability revealed a correlation between oxidative stress markers and specific fatty acids. Despite higher mean oxidative stress values for fish fed the $ \Sigma $ omega-3-rich diet, analysis of the individual heterogeneity in $ \Sigma $ omega-3 contents and oxidative stress management showed that individuals with high $ \Sigma $ omega-6 and low DHA had higher TBARS content. Consequently, high $ \Sigma $ omega-3/$ \Sigma $ omega-6 ratios were accompanied by lower oxidative stress levels. Some individuals appear to be more efficient at depositing omega-3s in tissue without the possible negative effects of dietary-induced increased oxidative stress on their health status. Additionally, this is not induced by higher antioxidant activity, at least for catalase and superoxide dismutase. The $ \Sigma $ omega-3/$ \Sigma $ omega-6 ratio could not however be the single determinant of oxidative stress susceptibility since at the same ratios, the TBARS content was higher in WW groups, suggesting that their concentration in tissues can also affect the lipid peroxidation process.

We observed a lower growth rate and higher impact of enriched ω-3 diets on the oxidative stress markers in Arctic charr compared to both brook charr and their hybrids. Considering the high commercial interest in Arctic charr, it appears that hybridization programmes might be a good strategy to improve productivity and health status of fish. Altogether, the present results are of particular interest for the aquaculture industry. Since consumer awareness concerning fish health is increasing (Ashley, 2007) and the consumption of $ \Sigma $ omega-3-rich food is highly encouraged (Calder and Yaqoob, 2009; Calder, 2014), selecting fish that have high $ \Sigma $ omega-3 content without the negative effects on health status through increased oxidative stress would be an interesting venue for breeding programmes. Furthermore, the relationship between TBARS and the $ \Sigma $ omega-3/$ \Sigma $ omega-6​ ratio, along with catalase, warrants further investigation to determine the significance of this ratio for the health status of individuals in natural populations.

Prior investigations from our group have established a relationship between the upper thermal tolerance limit (CTmax) in Arctic charr and both cardiac mitochondrial function and the management of oxidative stress (Christen *et al.,* 2018). Furthermore, we noted a negative correlation between CTmax and the relative abundance of ARA and EPA in the heart tissue of charr species and their hybrids. These observations collectively imply that the fatty acid composition of fish tissues plays a significant role in modulating the physiological stress response to temperature elevations. Based on these findings, it is evident that fatty acid profiles and the $ \Sigma $ omega-3/$ \Sigma $ omega-6​ ratio, when integrated with assessments of oxidative stress markers and antioxidant enzyme activities, offer promising tools for monitoring the health status and predicting the vulnerability and adaptive capacity of wild fish populations in the face of future climate change scenarios.

## Data Availability

The data that support the findings of this study are available on request from the corresponding author.
